# Numerical Study of Drift Influence on Diffusion Transport through the Hybrid Membrane

**DOI:** 10.3390/membranes12080788

**Published:** 2022-08-17

**Authors:** Monika Krasowska, Anna Strzelewicz, Gabriela Dudek, Michał Cieśla

**Affiliations:** 1 Faculty of Chemistry, Silesian University of Technology, Strzody 9, 44-100 Gliwice, Poland; 2 Institute of Theoretical Physics, Jagiellonian University, Łojasiewicza 11, 30-348 Kraków, Poland

**Keywords:** diffusion, drift, simulation, hybrid membrane, polymeric matrix, inorganic filler

## Abstract

Sodium alginate membranes filled with iron oxide nanoparticles consist of a mixture of organic and inorganic phases. This design offers the possibility to combine the polymer’s easy processability and superior separation performance. For a better understanding of the mechanisms of mixture separation, we analyze the diffusion motion of a particle in the hybrid membrane environment. We model structures of two-dimensional heterogenic membranes, which resemble real membrane structures, and then we simulate a random walk on them. We investigate how the additional action of drift changes the motion properties of the diffusing particles through the polymeric membrane filled with inorganic powder. We test the effect of two parameters: the distribution of obstacles (filling) in the membrane and the value of drift on the nature of diffusion. It appears that the synergy between drift, the diffusion, and the membrane structure affect the occurrence of the superdiffusive and subdiffusive character of particle motion as measured by the time-averaged mean square displacement. An important point is the observation that the strong drift supports subdiffusive motion as it increases the chances of particle trapping. Moreover, there exists the optimal value of drift, for which the transport through a membrane speeds up and does not cause trapping.

## 1. Introduction

The study of transport processes through polymeric membranes is an interesting but also a difficult and complex issue, especially if nonlinear and structure-morphology problems have to be addressed [1,2,3,4,5,6,7,8]. Research on the improvement of the efficiency of the separation processes can lead in two directions. In the first, researchers concentrate on materials, i.e., they are searching for new membrane materials or modifying existing ones [9,10,11,12,13]. In the second, the process conditions are investigated. Here, the focus is set on understanding the nature and details of transport and separation phenomena [10,12,14,15,16]. To date, several studies have investigated the use of biopolymers such as cellulose, chitosan, and alginate in the field of separation technology field [17,18,19,20,21,22,23,24,25]. Among others, alginate is a material that exhibits unique and useful properties. It is non-toxic and is considered as a prospective membrane material for the adsorption of dyes or metal ions and the separation of organic liquid mixtures. It can be used in a pristine or modified form. A substantial study in this area is the work of Dudek et al. [21,26], which involved alginate membranes with different contents of iron oxide nanoparticles and cross-linked by four different agents, in the process of ethanol dehydration. The study helped one to better understand how cross-linking agents and iron oxide powder content can change the transport properties. The results showed that the highest values of flux and the best separation properties are exhibited by membranes that contain 15 wt% of magnetite, crosslinked with phosphoric acid and calcium chloride. To better understand the mechanisms of mixture separation and its efficiency, we analyzed the diffusion motion of a particle in the membrane environment, in our previous paper [25]. We modeled the structures of two-dimensional heterogenic membranes, which resembled real membrane structures, and then we simulated a random walk on them. Brownian motion is a classic example of Markovian diffusion, where the variance of the particle position grows linearly in time [27,28]. If the particle’s displacements are independent and identically distributed according to a density with finite variance, the particle position follows the normal (Gaussian) distribution with the linearly growing variance. However, the experimental observations sometimes show deviations from the linear scaling of variance because of violations of assumptions regarding the independence or the boundedness of displacements. They can be a result of the influence of the structure properties of the environment in which the diffusion occurs. In such situations, the observed process is called anomalous diffusion [27,29,30,31,32,33,34]. The diffusion type is quantified using the mean square displacement (MSD), which can be calculated from the set of *N* trajectories $r_{i}\left(t\right)$:

(1) $$\langle \Delta r^{2}\rangle =\frac{1}{N}\sum_{i=1}^{N}{\left[r_{i}\left(t\right)-r\left(0\right)\right]}^{2}$$

The diffusion type is characterized by the exponent describing the growth of the MSD

(2) $$\langle \Delta r^{2}\rangle =D_{\alpha }t^{\alpha }$$

where α < 1 denotes the subdiffusion, while for α > 1 the superdiffusion is observed. Subdiffusion is typically observed when moving particles are trapped and the distribution of time inside a trap does not have a finite mean value. On the other hand, the standard mechanism of superdiffusion is caused by very long jumps of the diffusing particle. Here, the average value of the jump length is not defined. The limiting case of α = 1 corresponds to the standard diffusion, while α = 2 corresonds to the ballistic motion. Such a type of motion is caused, for example, by an external force, which causes a constant drift of moving particles. The discrimination of the diffusion type solely on the mean square displacement can be misleading [30]. Alternatively to the ensemble averaging, it is possible to calculate the time-averaged mean square displacement.

The main purpose of this study is to find, numerically, the influence of external drift on effective transport through the membrane. In previous studies, we observed that, in general, the presence of obstacles in the membrane can slow down the transport and cause subdiffusion [24,25]. On the other hand, a drift applied to a particle in an empty space accelerates its movement and follows to the ballistic motion. Therefore, the transport through the membrane with the external drift will be a result of these two competing effects. The obtained results may have an impact on optimizing transport in real membranes.

The structure of the paper takes the form of four main sections: Materials and Methods, Results, Discussion, and Conclusions. The membranes and the model are described in the Section 2 (Materials and Methods). Section 3 (Results) presents the findings of the research and graphs. Section 4 (Discussion) analyzes the results. The manuscript is closed with Conclusions (Section 5).

## 2. Materials and Methods

### 2.1. Real Membrane Preparation

**Figure 1:**
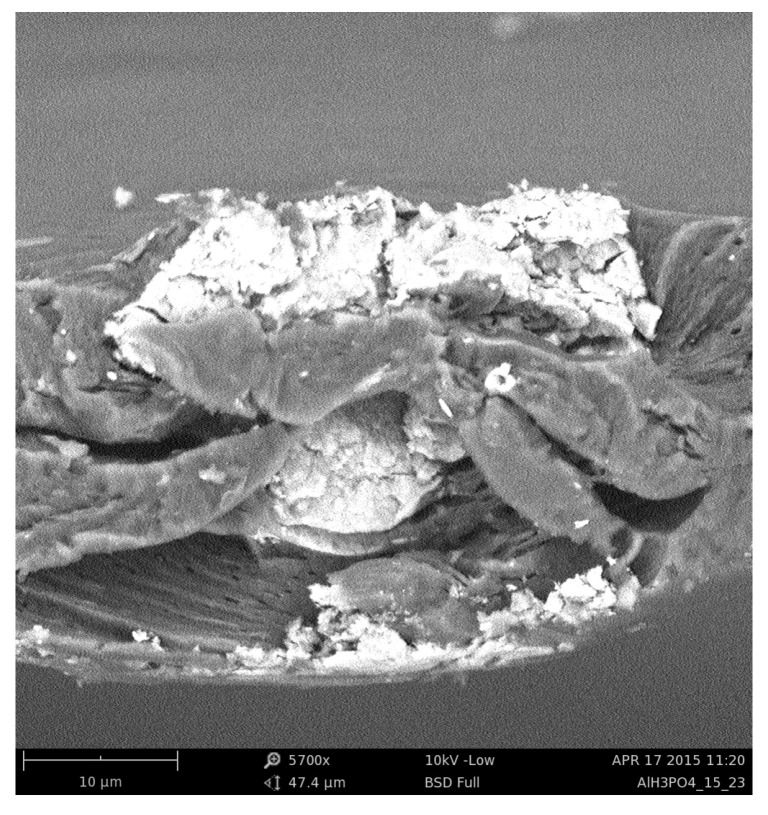
Image of an exemplary alginate membrane, cross-linked by phosphoric acid, containing 15 wt% of magnetite particles.

Sodium alginate membranes filled with iron oxide nanoparticles were prepared according to the scheme described in detail in Refs. [21,26]. Here, we provide only a brief description of the membrane preparation. Firstly, iron oxide nanoparticles were prepared. A mixture of FeCl${}_{6}$· 6H${}_{2}$O, sodium acetate, and ethylene glycol was stirred at 50 °C up to the time when a transparent solution was obtained. Then $2,2^{\prime }\left(ethylenedioxy\right)bis\left(ethylamine\right)$ was added to the resultant solution. The mixture was stirred and heated until ethylene glycol was evaporated, and the brown suspension was transformed into a black colloid. The resulting particles were subjected to magnetic decantation and were washed with distilled water, ethanol, and acetone. As a second step, sodium alginate membranes were prepared. A solution of dissolved sodium alginate in deionised water was mixed with an appropriate amount of magnetite nanoparticles. The mixture was then cast onto a glass plate and evaporated to dryness. Next, the membranes were cross-linked. In this paper, we work with membranes cross-linked with calcium chloride and phosphoric acid and filled with 15 wt % of magnetite. Images of cross-sections of the membranes were taken using a scanning electron microscope and saved in PNG format. Such images are the best representation of the actual morphology of the material. Figure 1 shows SEM image of an exemplary hybrid alginate membrane cross-linked by phosphoric acid containing 15 wt % of magnetite.

### 2.2. Binarisation and Morphology Analysis

**Figure 2:**
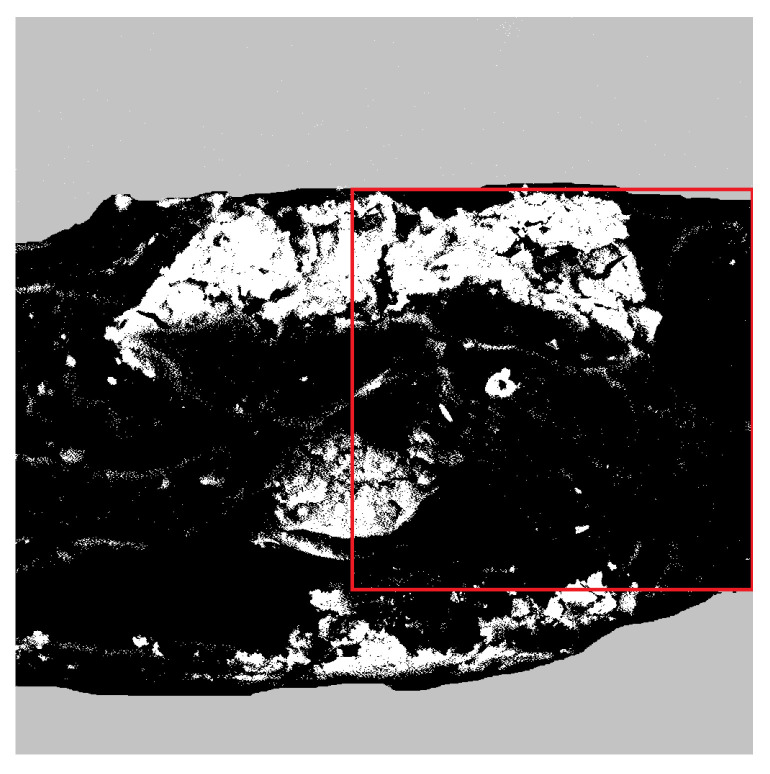
Image of exemplary alginate membrane, cross-linked by phosphoric acid, containing 15 wt% of magnetite particles-prepared for particle motion analysis. The area of 1024 × 1024 pixels used for further analysis is marked with a red square.

The two-dimensional SEM images of hybrid alginate membranes were digitized and converted to black and white pictures. The binarisation of exemplary Figure 1 is shown in Figure 2. The white regions correspond to obstacles, i.e., magnetite, while the black regions correspond to the alginate matrix.

**Figure 3:**
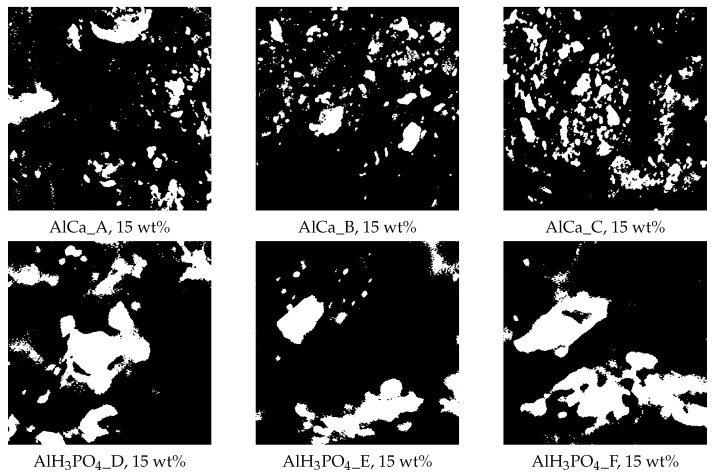
Exemplary images of alginate membranes, i.e., cross-linked by calcium chloride and phosphoric acid, containing 15 wt% magnetite particles, prepared for numerical simulation and analysis. Structural parameters: AlCa_A (ρ = 0.89, $d_{f}$ = 1.9751, ΔD = 0.3974B; AlCa_B (ρ = 0.87, $d_{f}$ = 1.9809, ΔD = 0.3205); AlCa_C (ρ = 0.84, $d_{f}$ = 1.9711, ΔD = 0.2310); AlH${}_{3}$PO${}_{4}$_D (ρ = 0.79, $d_{f}$ = 1.9531, ΔD = 2.0629); AlH${}_{3}$PO${}_{4}$_E (ρ = 0.66, $d_{f}$ = 1.9137, ΔD = 1.8077); and AlH${}_{3}$PO${}_{4}$_F (ρ = 0.77, $d_{f}$ = 1.9462, ΔD = 1.2272).

The morphological and diffusional analysis of the real membranes was based on the images of hybrid alginate membranes, cross-linked by phosphoric acid or calcium chloride, containing 15 wt% of magnetite particles and shown in Figure 3. These are images cut to a size of 1024 × 1024 pixels from a binarized SEM image of a real membrane. The morphology of each membrane was characterized using the following parameters:

- The observed amount of polymer matrix ρ, which is defined as the ratio of the polymer matrix area visible in the picture (black regions) to the total image area,
- The fractal dimension of polymer matrix $d_{f}$,
- The degree of multifractality ΔD.

The surface characterization is described in detail in [24,25].

### 2.3. Artificial Membranes

**Figure 4:**
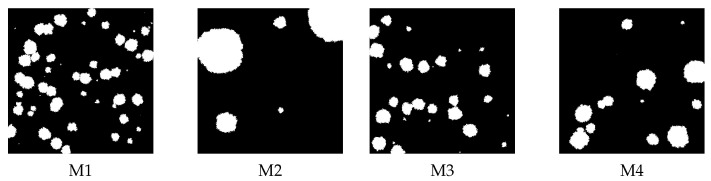
The artificial membrane structures with different structural parameters: M1 (ρ = 0.85, $d_{f}$ = 1.9707, ΔD = 0.3492); M2 (ρ = 0.85, $d_{f}$ = 1.9672, ΔD = 1.5094); M3 (ρ = 0.9, $d_{f}$ = 1.9830, ΔD = 0.2906); and M4 (ρ = 0.9, $d_{f}$ = 1.9790, ΔD = 1.8948).

Since the morphological properties of real membranes are hard to control during membrane preparation, to determine important morphological factors for particle transport, we generated some images of artificial membranes that differ in the fraction of obstacle density or its size distribution. The images of structure of artificial membranes are generated using the method described in Refs. [24,25] and are presented in Figure 4.

The structures of artificial membranes are represented by black and white images of size: 1024 × 1024 pixels. The black regions correspond to the polymer matrix as for the structures of the real membranes and are available for diffusing particles. Artificial membranes are generated with prescribed parameters, such as the color saturation ratio ρ, which corresponds to the ratio of the number of filler particles added to the polymeric matrix and is reflected by the relation of white pixels to black pixels in the image. The generated textures were also analyzed in the same way as the real membranes (see Section 2.4), and the same parameters were determined for all created structures.

### 2.4. Diffusion Model

The diffusion through the membrane is studied numerically. A trial point-size particle is placed in a random black pixel of the image, and it undergoes Brownian motion with a drift. The single movement is determined as follows:

- The jump length ξ is selected randomly according to Gaussian probability distribution of zero mean and $\sigma^{2}$ variance;
- The direction of the above jump is selected randomly from the uniform probability distribution in the interval [0,2π)
- The constant term corresponding to the drift $\left[\Lambda_{x},\Lambda_{y}\right]$ is added to the previously determined movement.
- The position of the particle is updated only if the movement does not end in or intersect an obstacle.

This scheme reproduces THE overdamped motion of the particle, which is typical for colloids and solutions. It can be expressed by the following equation:

(3) $$\frac{dr}{dt}=\Lambda +\xi \left(t\right),$$

where *r* denotes position of the particle. Note that the last point of the above-described scheme could be confusing because it might seem that the skipping of such illegal move could be non-physical. One can conclude that such a tracer could be reflected or at least stopped next to the obstacle surface. On the other hand, for a one-dimensional well, it has been proven that such a numerical approach reproduces an analytically obtained distribution of particle positions [35].

We adjusted the laboratory coordinates frame to make the drift parallel to its *x* axis; thus, $\Lambda_{x}=\Lambda$ and $\Lambda_{y}=0$. Thus, the jumps are fully determined by two parameters: σ, and Λ. In the simulation, we used periodic boundary conditions for diffusing particles to not limit their movement by the boundaries of the image.

The trajectories of the particles are then analyzed using Equation (2):

(4) $$\ln\langle \Delta r^{2}\rangle =\ln D_{\alpha }+\alpha \ln t.$$

The linear fit to the measured data $\left(t,\langle \Delta r^{2}\rangle \right)$ allows one to obtain the diffusion constant *D* and the exponent α.

Typically, we used N=1000 independent random trajectories of the number of steps $t=10^{6}$. The Brownian motion, across the whole simulation, was characterized by σ=0.01. Distances are measured in pixels–one pixel corresponds approximately to 5 nm. Time is measured in the time-steps mentioned above.

## 3. Results

### 3.1. Real Membranes

**Figure 5:**
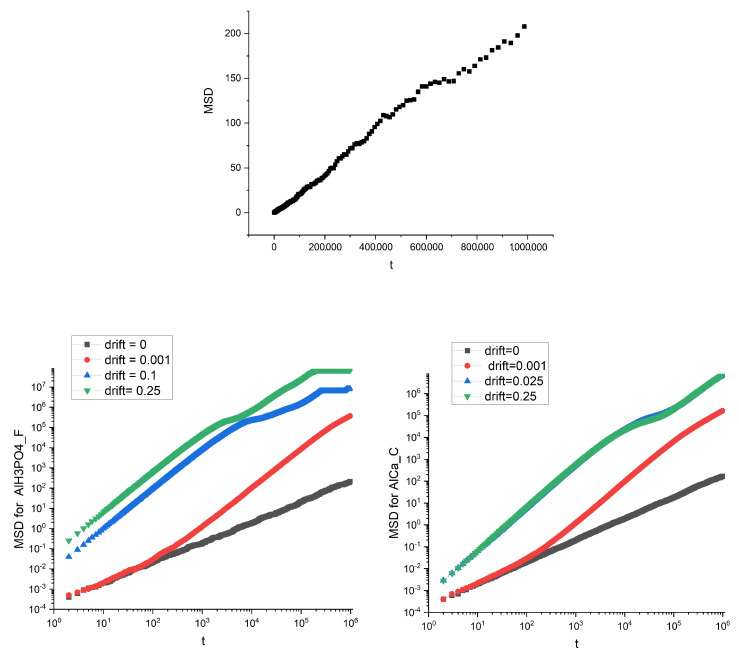
The mean square displacement dependence on time for the image of AlH${}_{3}$PO${}_{4}$_F membrane for Λ=0 (**top panel**). Linear dependence of MSD on t denotes standard diffusion: α=0. Bottom panel are for AlH${}_{3}$PO${}_{4}$_F membrane (**left**) and AlCa_C membrane (**right**) and various drifts. Log-log scale is used for convenience.

The diffusive motion of the particles, both in the presence and the absence of drift, was simulated on the structures that are the images of real membranes: the presence of obstacles in the membrane (magnetite in alginate matrix) and the action of the drift influence on the mean square displacements. Example plots of MSD dependence on time for two different membranes and several values of drift (i.e., Λ=0.001, 0.1, 0.25 and drift absence Λ=0) are shown in Figure 5.

Not surprisingly, the drift speeds up the transport measured using MSD; however, we can also observe a saturation, especially for the Al H${}_{3}$PO${}_{4}$_F membrane for higher values of the drift.

**Figure 6:**
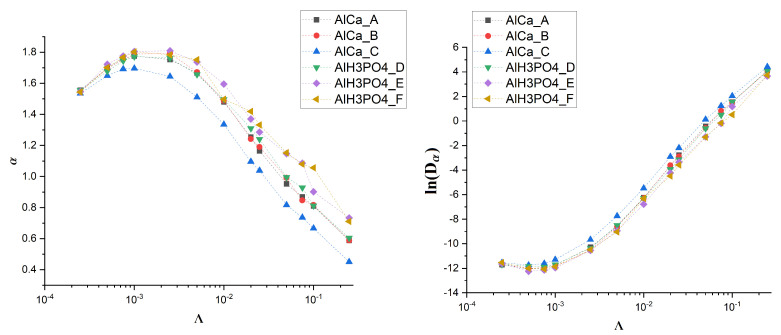
Dependence of α (**left**) and $\ln D_{\alpha }$ (**right**) on drift for various membranes.

To obtain deeper insight into the diffusion properties, we estimate the diffusion coefficient $D_{\alpha }$ and the exponent α (see Equation (2)). The results are presented in Figure 6.

Without the drift, the observed value of the exponent α is approximately equal to 1 (see Figure 5). A small amount of drift Λ≈0.001 causes its increase up to 1.8, which is almost the value characteristic for ballistic motion. However, the further growth of the drift causes the exponent to decrease, and for Λ>0.05 the subdiffusive regime begins as α drops significantly below 1. The diffusion coefficient $D_{\alpha }$ behaves in an opposite way. After reaching the shallow minimum for Λ≈0.0005, $D_{\alpha }$ begins to grow significantly.

It is noteworthy that all these observations are common among all studied membranes, which suggests that the effect is universal and does not depend on the details of membrane morphology. To check this, we decide to generate some artificial membranes that differ in the fraction of obstacle density and their size distribution (see Figure 4).

### 3.2. Artificial Membranes

**Figure 7:**
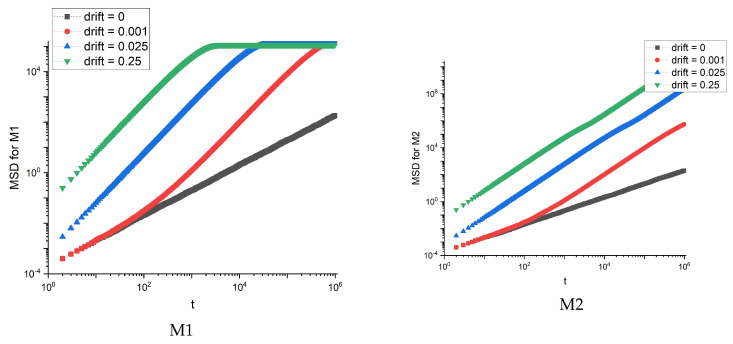
MSD dependence on time for artificial membranes M1 and M2.

Similar diffusion simulations in the absence (Λ=0) and presence of drift (Λ=0.001, 0.025, 0.25), as for the real membranes, were performed for the artificial structures. The mean square displacements for the artificial membranes (Figure 4) are shown in Figure 7.

**Figure 8:**
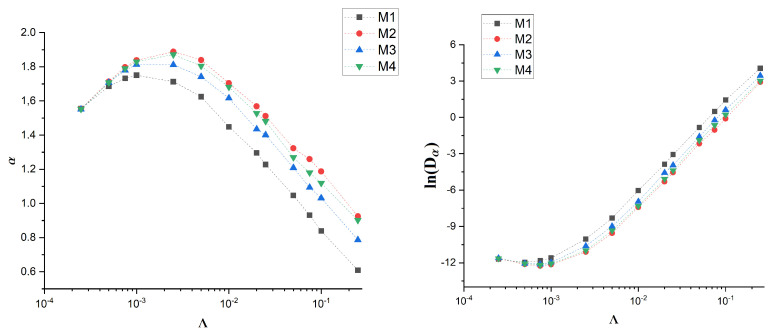
Dependence of α (**left**) and $\ln D_{\alpha }$ (**right**) on drift for artificial membranes M1-4.

For the M1 membrane, we observe the saturation of MSD, which occurs faster for stronger drift. For other membranes, the dependence of MSD on time looks the same as for M2 membrane. The saturation is also reflected in the effective diffusion exponent α and diffusion coefficient $D_{\alpha }$—see Figure 8.

The effective exponent α for M1 membrane is the lowest. The effect is stronger with the increase of drift Λ. On the other hand, the diffusion coefficient $D_{\alpha }$ is the highest for M1. These observations are qualitatively the same as for real membranes with only a slight quantitative difference—for artificial membranes the effective exponent α is, in general, slightly larger.

For convenience, all values are collected in the Appendix A in Table A1 and Table A2 for real membranes, and Table A3 and Table A4 for artificial ones.

## 4. Discussion

**Figure 9:**
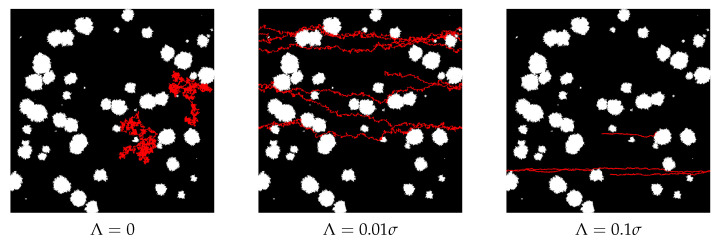
Exemplary trajectories of two diffusing particles without drift Λ=0, and with two different values of drift Λ=0.01σ and Λ=0.1σ. Here, σ=0.05 and trajectory length $t=10^{7}$. Note the periodic boundary conditions.

To illustrate and discuss the influence of drift on the diffusing particle, we show two exemplary trajectories for three different values of drift—see Figure 9.

For Λ=0, we can observe the standard Brownian motion restricted by the presence of obstacles (left panel in Figure 9). The appearance of drift is clearly visible as the trajectories become horizontal. When the drift is relatively small—as in the middle panel in Figure 9—the transport speeds up, but the random diffusion is strong enough to avoid the obstacles. However, when the drift becomes stronger, some trajectories can be pushed to the edge of an obstacle, and Gaussian diffusion is too weak to move a particle away from the obstacle and allow its avoidance. It is clearly visible in the upper trajectory in the right panel of Figure 9. In other words, when the drift is too large, it can even stop the transport, see Figure 9. This effect was observed and discussed in detail in Ref. [31].

The above observation explains the results presented in the former section. Small drift can convert diffusion into ballistic motion, which is much more effective as a source of transport than Brownian diffusion. This explains the growth of the effective diffusion exponent α for relatively small Λ. For higher Λ, some trajectories are trapped, which causes a decrease of α. When the drift and the number of obstacles are high enough, all trajectories can be trapped, and then we observe the saturation of MSD.

Because in our system the drift acts along the horizontal axis, the most important factors that affect the particle movement are that the fraction of horizontal paths that does not intersect an obstacle and that it allows for unobstructed transport. When this fraction is small, as in the M1 membrane, the moving particles are stuck at the obstacles and therefore the saturation of MSD is observed and the transport becomes subdiffusive. The same observation refers to the AlCa_C and AlH${}_{3}$PO${}_{4}$_D membranes. Note that the number of obstacles or there size distribution is of secondary importance. Moreover, all the morphology descriptors introduced in the Materials and Methods section are irrelevant, which contrasts with previous observations made for the absence of the drift [24,25].

## 5. Conclusions

Anomalous transport in crowded environments has attracted the attention of researchers for several years. To date, theoretical tools have been developed to understand the non-linear behavior of MSD in systems with anomalous diffusion. The present study was designed to determine the effect of the action of drift on the diffusing particles in the polymeric membrane with filler. The action of a constant drift influences moving particles. These particles are trapped on the obstacles present in the membrane.

The drift introduces a preferred direction of motion, which is further altered by interactions with obstacles in membrane matrix. Although drift in general speeds up the transport, it can also lead to subdiffusive motion as it increases the chances of particle trapping at the surface of the obstacle. Overall, this study suggests that there is optimal value of drift, which speeds up transport and does not lead to particle trapping. In our case, it corresponds to Λ≈0.01σ−0.1σ; thus the constant drift should be ten times smaller than the random Brownian movement. This observation can lead to direct applications as the diffusion constant can be measured experimentally or approximated by Einstein–Smoluchowski or Stokes–Einstein relations

(5) $$\begin{array}{c} D=\frac{\mu k_{B}T}{q},\\ D=\frac{k_{B}T}{6\pi \eta r}, \end{array}$$

where μ is mobility of particle with charge *q*, η is friction constant, *r* is radius of spherical particle, $k_{B}$ is Boltzmann constant, and *T* is temperature. For Gaussian diffusion $\sigma =\sqrt{2Dt}$, which allows one to determine the optimal drift range. On the other hand, due to the simplicity of our model, these results should be treated as a starting point for more detailed analysis. Further research could also be conducted to determine the influence of drift on organic mixture separation, but it requires greater effort to plan experiments in the laboratory.

## Data Availability

The numerical data, analyzed images, and necessary software are available upon reasonable request.

## Abbreviations

The following abbreviations are used in this manuscript:

AlCa	Alginate membrane cross-linked by calcium chloride with 15 wt% of magnetite particles
AlH${}_{3}$PO${}_{4}$	Alginate membrane cross-linked by phosphoric acid with 15 wt% of magnetite particles
MSD	Mean square displacement

## Appendix A

**Table A1:** Transport properties of alginate membrane cross-linked by calcium chloride, containing 15 wt% of magnetite particles.

Membrane	Λ	α	${\mathrm{lnD}}_{\alpha }$
AlCa_A	0.00000	1.0027 ± 0.0006	−8.9173 ± 0.0057
AlCa_A	0.00025	1.5548 ± 0.0108	−11.7190 ± 0.0965
AlCa_A	0.00050	1.6974 ± 0.0091	−12.0403 ± 0.0813
AlCa_A	0.00075	1.7609 ± 0.0075	−12.0175 ± 0.0671
AlCa_A	0.00100	1.7752 ± 0.0067	−11.7494 ± 0.0601
AlCa_A	0.00250	1.7527 ± 0.0099	−10.2851 ± 0.0879
AlCa_A	0.00500	1.6665 ± 0.0137	−8.5712 ± 0.1222
AlCa_A	0.01000	1.4797 ± 0.0187	−6.2336 ± 0.1662
AlCa_A	0.02000	1.2534 ± 0.0228	−3.6803 ± 0.2029
AlCa_A	0.02500	1.1647 ± 0.0243	−2.7738 ± 0.2161
AlCa_A	0.05000	0.9528 ± 0.0256	−0.4219 ± 0.2276
AlCa_A	0.07500	0.8689 ± 0.0253	0.7349 ± 0.2251
AlCa_A	0.10000	0.8096 ± 0.0251	1.5445 ± 0.2234
AlCa_A	0.25000	0.5873 ± 0.0229	4.1089 ± 0.2040
AlCa_B	0.00000	1.0016 ± 0.0006	−8.8763 ± 0.0058
AlCa_B	0.00025	1.5491 ± 0.0105	−11.6427 ± 0.0938
AlCa_B	0.00050	1.6946 ± 0.0092	−12.0225 ± 0.0820
AlCa_B	0.00075	1.7529 ± 0.0073	−11.9371 ± 0.0655
AlCa_B	0.00100	1.7866 ± 0.0065	−11.8214 ± 0.0583
AlCa_B	0.00250	1.7926 ± 0.0084	−10.5556 ± 0.0746
AlCa_B	0.00500	1.6718 ± 0.0132	−8.5978 ± 0.1176
AlCa_B	0.01000	1.4880 ± 0.0180	−6.2783 ± 0.1606
AlCa_B	0.02000	1.2414 ± 0.0234	−3.6113 ± 0.2080
AlCa_B	0.02500	1.1898 ± 0.0243	−2.8969 ± 0.2161
AlCa_B	0.05000	0.9915 ± 0.0256	−0.5834 ± 0.2274
AlCa_B	0.07500	0.8467 ± 0.0253	0.8250 ± 0.2249
AlCa_B	0.10000	0.8166 ± 0.0251	1.5149 ± 0.2233
AlCa_B	0.25000	0.5888 ± 0.0230	4.1083 ± 0.2046
AlCa_C	0.00000	0.9872 ± 0.0006	−8.8056 ± 0.0061
AlCa_C	0.00025	1.5352 ± 0.0103	−11.5435 ± 0.0918
AlCa_C	0.00050	1.6480 ± 0.0083	−11.7404 ± 0.0740
AlCa_C	0.00075	1.6915 ± 0.0075	−11.6050 ± 0.0665
AlCa_C	0.00100	1.6963 ± 0.0084	−11.2913 ± 0.0745
AlCa_C	0.00250	1.6440 ± 0.0129	−9.6653 ± 0.1145
AlCa_C	0.00500	1.5111 ± 0.0173	−7.7364 ± 0.1541
AlCa_C	0.01000	1.3353 ± 0.0211	−5.4783 ± 0.1882
AlCa_C	0.02000	1.0957 ± 0.0247	−2.9066 ± 0.2198
AlCa_C	0.02500	1.0300 ± 0.0253	−2.1543 ± 0.2248
AlCa_C	0.05000	0.8166 ± 0.0251	0.1305 ± 0.2234
AlCa_C	0.07500	0.7366 ± 0.0246	1.2344 ± 0.2188
AlCa_C	0.10000	0.6669 ± 0.0240	2.0422 ± 0.2131
AlCa_C	0.25000	0.4507 ± 0.0207	4.4201 ± 0.1838

**Table A2:** Transport properties of alginate membrane cross-linked by phosphoric acid, containing 15 wt% of magnetite particles.

Membrane	Λ	α	${\mathrm{lnD}}_{\alpha }$
$AlH_{3}PO_{4}\_D$	0.00000	1.0075 ± 0.0020	−8.9991 ± 0.0186
$AlH_{3}PO_{4}\_D$	0.00025	1.5542 ± 0.0109	−11.6921 ± 0.0971
$AlH_{3}PO_{4}\_D$	0.00030	1.5902 ± 0.0103	−11.7740 ± 0.0917
$AlH_{3}PO_{4}\_D$	0.00040	1.6483 ± 0.0106	−11.8981 ± 0.0947
$AlH_{3}PO_{4}\_D$	0.00050	1.6777 ± 0.0093	−11.8377 ± 0.0833
$AlH_{3}PO_{4}\_D$	0.00075	1.7442 ± 0.0073	−11.9039 ± 0.0651
$AlH_{3}PO_{4}\_D$	0.00100	1.7712 ± 0.0074	−11.6999 ± 0.6572
$AlH_{3}PO_{4}\_D$	0.00250	1.7620 ± 0.0107	−10.3687 ± 0.0953
$AlH_{3}PO_{4}\_D$	0.00500	1.6567 ± 0.0148	−8.4944 ± 0.1316
$AlH_{3}PO_{4}\_D$	0.01000	1.4961 ± 0.0199	−6.2902 ± 0.1770
$AlH_{3}PO_{4}\_D$	0.02000	1.3099 ± 0.0233	−3.9347 ± 0.2070
$AlH_{3}PO_{4}\_D$	0.02500	1.2404 ± 0.0242	−3.1323 ± 0.2155
$AlH_{3}PO_{4}\_D$	0.05000	0.9949 ± 0.0253	−0.6088 ± 0.2250
$AlH_{3}PO_{4}\_D$	0.07500	0.9289 ± 0.0256	0.4937 ± 0.2273
$AlH_{3}PO_{4}\_D$	0.10000	0.8115 ± 0.0251	1.5388 ± 0.2235
$AlH_{3}PO_{4}\_D$	0.25000	0.6040 ± 0.0233	4.0647 ± 0.2066
$AlH_{3}PO_{4}\_D$	0.50000	0.4517 ± 0.0207	5.8110 ± 0.7370
$AlH_{3}PO_{4}\_E$	0.00000	1.0018 ± 0.0019	−8.9305 ± 0.0177
$AlH_{3}PO_{4}\_E$	0.00025	1.5450 ± 0.0119	−11.6071 ± 0.1061
$AlH_{3}PO_{4}\_E$	0.00030	1.6047 ± 0.0104	−11.8923 ± 0.0926
$AlH_{3}PO_{4}\_E$	0.00040	1.6674 ± 0.0104	−12.0907 ± 0.0926
$AlH_{3}PO_{4}\_E$	0.00050	1.7226 ± 0.0096	−12.2484 ± 0.0855
$AlH_{3}PO_{4}\_E$	0.00075	1.7749 ± 0.0080	−12.1502 ± 0.0718
$AlH_{3}PO_{4}\_E$	0.00100	1.8042 ± 0.0071	−11.9647 ± 0.0638
$AlH_{3}PO_{4}\_E$	0.00250	1.8087 ± 0.0084	−10.5423 ± 0.0748
$AlH_{3}PO_{4}\_E$	0.00500	1.7371 ± 0.0133	−8.9492 ± 0.1186
$AlH_{3}PO_{4}\_E$	0.01000	1.5943 ± 0.0177	−6.7806 ± 0.1574
$AlH_{3}PO_{4}\_E$	0.02000	1.3698 ± 0.0222	−4.2374 ± 0.1979
$AlH_{3}PO_{4}\_E$	0.02500	1.2861 ± 0.0237	−3.3489 ± 0.2107
$AlH_{3}PO_{4}\_E$	0.05000	1.1470 ± 0.0251	−1.2961 ± 0.2234
$AlH_{3}PO_{4}\_E$	0.07500	1.0860 ± 0.0254	−0.1959 ± 0.2257
$AlH_{3}PO_{4}\_E$	0.10000	0.9023 ± 0.0255	1.1783 ± 0.2268
$AlH_{3}PO_{4}\_E$	0.25000	0.7335 ± 0.0246	3.6554 ± 0.2188
$AlH_{3}PO_{4}\_E$	0.50000	0.5296 ± 0.0221	5.6490 ± 0.1969
$AlH_{3}PO_{4}\_F$	0.00000	1.0038 ± 0.0019	−8.9172 ± 0.0174
$AlH_{3}PO_{4}\_F$	0.00025	1.5435 ± 0.0100	−11.5307 ± 0.0892
$AlH_{3}PO_{4}\_F$	0.00030	1.5946 ± 0.0099	−11.7422 ± 0.0882
$AlH_{3}PO_{4}\_F$	0.00040	1.6585 ± 0.0101	−11.9446 ± 0.0901
$AlH_{3}PO_{4}\_F$	0.00045	1.6800 ± 0.0099	−11.9983 ± 0.0881
$AlH_{3}PO_{4}\_F$	0.00050	1.7015 ± 0.0093	−12.0099 ± 0.0827
$AlH_{3}PO_{4}\_F$	0.00075	1.7664 ± 0.0079	−12.0747 ± 0.0701
$AlH_{3}PO_{4}\_F$	0.00100	1.8016 ± 0.0062	−11.8638 ± 0.0552
$AlH_{3}PO_{4}\_F$	0.00250	1.7829 ± 0.0097	−10.4806 ± 0.0862
$AlH_{3}PO_{4}\_F$	0.00500	1.7512 ± 0.0124	−9.0391 ± 0.1109
$AlH_{3}PO_{4}\_F$	0.01000	1.4974 ± 0.0205	−6.3091 ± 0.1824
$AlH_{3}PO_{4}\_F$	0.02000	1.4187 ± 0.0221	−4.4949 ± 0.1967
$AlH_{3}PO_{4}\_F$	0.02500	1.3322 ± 0.0234	−3.5878 ± 0.2079
$AlH_{3}PO_{4}\_F$	0.05000	1.1523 ± 0.0251	−1.3182 ± 0.2230
$AlH_{3}PO_{4}\_F$	0.07500	1.0796 ± 0.0254	−0.1681 ± 0.2261
$AlH_{3}PO_{4}\_F$	0.10000	1.0560 ± 0.0254	0.5146 ± 0.2259
$AlH_{3}PO_{4}\_F$	0.25000	0.7108 ± 0.0244	3.7301 ± 0.2170
$AlH_{3}PO_{4}\_F$	0.50000	0.5531 ± 0.0225	5.5903 ± 0.2000

**Table A3:** Transport properties of artificial membranes with different structural parameters: M1 (ρ = 0.85, $d_{f}$ = 1.9707, and ΔD = 0.3492); M2 (ρ = 0.85, $d_{f}$ = 1.9672, and ΔD = 1.5094).

Membrane	Λ	α	${\mathrm{lnD}}_{\alpha }$
M1	0.00000	0.9898 ± 0.0006	−8.8089 ± 0.0054
M1	0.00025	1.5547 ± 0.0108	−11.7079 ± 0.0964
M1	0.00050	1.6855 ± 0.0089	−11.9618 ± 0.0798
M1	0.00075	1.7323 ± 0.0079	−11.8228 ± 0.0707
M1	0.00100	1.7505 ± 0.0078	−11.5999 ± 0.0699
M1	0.00250	1.7128 ± 0.0126	−10.0363 ± 0.1122
M1	0.00500	1.6247 ± 0.0167	−8.3096 ± 0.1489
M1	0.01000	1.4487 ± 0.0208	−6.0322 ± 0.1849
M1	0.02000	1.2968 ± 0.0234	−3.8709 ± 0.2085
M1	0.02500	1.2275 ± 0.0243	−3.0686 ± 0.2159
M1	0.05000	1.0470 ± 0.0255	−0.8288 ± 0.2268
M1	0.07500	0.9315 ± 0.0256	0.4812 ± 0.2273
M1	0.10000	0.8393 ± 0.0253	1.4301 ± 0.2248
M1	0.25000	0.6090 ± 0.0233	4.0524 ± 0.2071
M2	0.00000	1.0036 ± 0.0006	−8.9092 ± 0.0060
M2	0.00025	1.5535 ± 0.0108	−11.6600 ± 0.0958
M2	0.00050	1.7138 ± 0.0094	−12.1198 ± 0.0843
M2	0.00075	1.7980 ± 0.0082	−12.2650 ± 0.0736
M2	0.00100	1.8383 ± 0.0068	−12.1455 ± 0.0611
M2	0.00250	1.8882 ± 0.0059	−11.0998 ± 0.0531
M2	0.00500	1.8397 ± 0.0099	−9.5582 ± 0.0879
M2	0.01000	1.7040 ± 0.0155	−7.4194 ± 0.1383
M2	0.02000	1.5688 ± 0.0194	−5.2968 ± 0.1724
M2	0.02500	1.5120 ± 0.0206	−4.5355 ± 0.1833
M2	0.05000	1.3232 ± 0.0236	−2.1600 ± 0.2100
M2	0.07500	1.2594 ± 0.0243	−1.0291 ± 0.2161
M2	0.10000	1.1875 ± 0.0249	−0.1003 ± 0.2213
M2	0.25000	0.9250 ± 0.0256	2.9178 ± 0.2273

**Table A4:** Transport properties of artificial membranes with different structural parameters: M3 (ρ = 0.9, $d_{f}$ = 1.9830, and ΔD = 0.2906); M4 (ρ = 0.9, $d_{f}$ = 1.9790, and ΔD = 1.8948).

Membrane	Λ	α	${\mathrm{lnD}}_{\alpha }$
M3	0.00000	1.0008±0.0007	−8.8996 ± 0.0068
M3	0.00025	1.5509 ± 0.0107	−11.6224 ± 0.0950
M3	0.00050	1.7085 ± 0.0092	−12.0638 ± 0.0824
M3	0.00075	1.7791 ± 0.0079	−12.1137 ± 0.0708
M3	0.00100	1.8130 ± 0.0066	−11.9707 ± 0.0593
M3	0.00250	1.8119 ± 0.0090	−10.6145 ± 0.0804
M3	0.00500	1.7420 ± 0.0136	−8.9810 ± 0.1210
M3	0.01000	1.6163 ± 0.0177	−6.9412 ± 0.1578
M3	0.02000	1.4346 ± 0.0218	−4.5740 ± 0.1938
M3	0.02500	1.4006 ± 0.0223	−3.9472 ± 0.1985
M3	0.05000	1.2094 ± 0.0247	−1.5942 ± 0.2199
M3	0.07500	1.0931 ± 0.0254	−0.2297 ± 0.2258
M3	0.10000	1.0316 ± 0.0255	0.6248 ± 0.2272
M3	0.25000	0.7872 ± 0.0250	3.4615 ± 0.2223
M4	0.00000	1.0006 ± 0.0005	−8.8927 ± 0.0050
M4	0.00025	1.5524 ± 0.0107	−11.6419 ± 0.0957
M4	0.00050	1.7074 ± 0.0095	−12.0586 ± 0.0845
M4	0.00075	1.7903 ± 0.0082	−12.1917 ± 0.0730
M4	0.00100	1.8298 ± 0.0066	−12.0739 ± 0.0594
M4	0.00250	1.8712 ± 0.0064	−10.9861 ± 0.0573
M4	0.00500	1.8047 ± 0.0107	−9.3423 ± 0.0952
M4	0.01000	1.6816 ± 0.0158	−7.3097 ± 0.1409
M4	0.02000	1.5286 ± 0.0199	−5.0806 ± 0.1771
M4	0.02500	1.4812 ± 0.0210	−4.3725 ± 0.1872
M4	0.05000	1.2689 ± 0.0242	−1.8885 ± 0.2152
M4	0.07500	1.1801 ± 0.0249	−0.6404 ± 0.2218
M4	0.10000	1.1190 ± 0.0253	0.2243 ± 0.2249
M4	0.25000	0.9015 ± 0.0255	3.0148 ± 0.2268
